# Anticancer Properties of Lamellarins

**DOI:** 10.3390/md13031105

**Published:** 2015-02-19

**Authors:** Christian Bailly

**Affiliations:** Pierre Fabre Research Institute, Research & Development Center, 3 Avenue Hubert Curien, BP 13562, 31035 Toulouse cedex 1, France; E-Mail: christian.bailly@pierre-fabre.com; Tel.: +33-534-506-399

**Keywords:** lamellarins, marine natural products, cancer, topoisomerase I, mitochondria, drug synthesis

## Abstract

In 1985 the first lamellarins were isolated from a small oceanic sea snail. Today, more than 50 lamellarins have been inventoried and numerous derivatives synthesized and tested as antiviral or anticancer agents. The lead compound in the family is lamellarin D, characterized as a potent inhibitor of both nuclear and mitochondrial topoisomerase I but also capable of directly interfering with mitochondria to trigger cancer cell death. The pharmacology and chemistry of lamellarins are discussed here and the mechanistic portrait of lamellarin D is detailed. Lamellarins frequently serve as a starting point in the design of anticancer compounds. Extensive efforts have been devoted to create novel structures as well as to improve synthetic methods, leading to lamellarins and related pyrrole-derived marine alkaloids.

## 1. The Lamellarin Class of Marine Alkaloids

Marine prosobranch mollusc species are abundant and usually actively predate on bivalves, using toxins in food procurement. For example, the red whelk *Neptunea antiqua* (family Buccinidae)—a sublittoral species that occurs in the cold temperate waters of the eastern Atlantic boreal region—uses its salivary gland neurotoxin tetramine for hunting and feeding [1]. These molluscs rely on bioactive compounds for feeding but also as defensive elements to escape predation. Due to their extraordinary capacity to produce a variety of complex chemical substances, marine organisms, and molluscs in particular, have become a hotspot of research over the past twenty years [2]. Indole and pyrrole alkaloids, such as topsentin [3,4], tambjamine D [5], spongiacidin C [6], the discorhabdines [7], bear therapeutic potential and are frequently considered as a source of anticancer drugs [8,9]. Chemical modifications of natural products may lead to innovative drugs endowed with potent antitumor activities. This is the case for the two synthetic iminoquinones derivatives BA-TPQ and its fluoro derivative FBA-TPQ, analogues of the alkaloids makaluvamines, which display significant antitumor activities *in vitro* and *in vivo* on different xenograft models [10,11,12,13,14]. Like the related compounds batzellines, makaluvamines (isolated from sponges of the genus *Zyzzya*), they were initially characterized as topoisomerase II inhibitors able to produce protein-linked DNA double-strand breaks [15,16] but in fact the underlying mechanism of action of FBA-TPQ is much more complex and not fully understood. The compounds seem to activate the ZAK-MKK4-JNK-TGFβ signaling cascade as a molecular target for their anticancer activity [17]. A similar trend can be evoked for another interesting series of pyrroloiminoquinones, the tsitsikammamines initially isolated from South African latrunculid sponges and characterized as topoisomerase I inhibitors [18,19]. However, a recent study refers to a completely different mechanism of action for a tsitsikammamine A analogue, with a specific inhibition of indoleamine 2,3-dioxygenase (IDO1), an emerging immuno-therapeutic target for the treatment of cancer [20]. This example illustrates the difficulty to delineate the molecular mechanism of action of natural products and to establish structure-activity relationships. The literature is rich in examples of old drugs with a new mechanism of action [21,22]. In most cases, our knowledge of the mechanism of action of natural products remains fragmentary and this is certainly also the case for the lamellarins.

**Figure 1:**
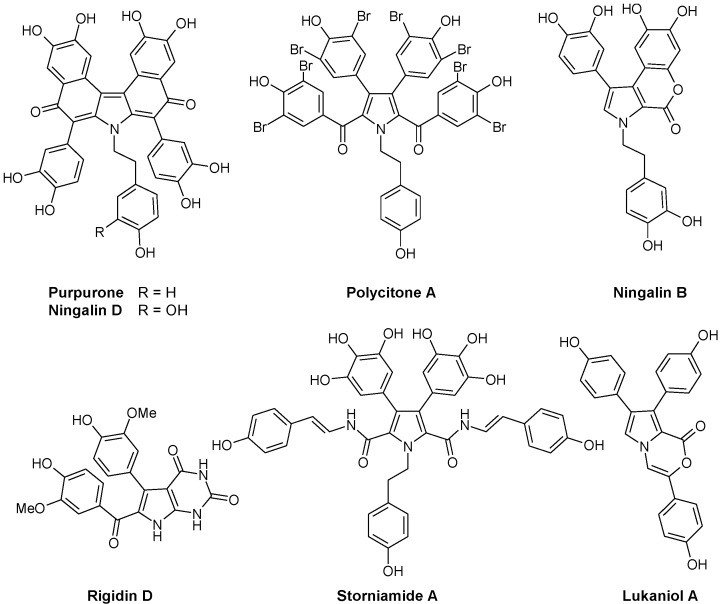
Selected examples of pyrrole marine alkaloids.

Another group of marine alkaloids of pharmacological interest derives from the amino acid 3,4-dihydroxyphenylalanine (or 2-amino-3-(3',4'-dihydroxyphenyl) propionic acid, L-DOPA) which is recognized as a key precursor to a broad variety of structurally unique alkaloids in marine invertebrates [23]. DOPA is at the origin of naturally occurring polyaromatic molecules with a pyrrole core, differing in the substitution pattern and the arrangement of the rings. At least five structural subtypes have been described, all bearing a central pyrrole surrounded by aromatic units (Figure 1):

- Lukianols with a *N*-alkylpyrrolecarboxylic acid core.
- Rigidins, antimitotic pyrrolopyrimidine alkaloids that inhibit tubulin polymerization and disorganize microtubules [24,25,26,27].
- Polycitrins and polycitones, a small group with a rare molecular skeleton. Polycitone A, isolated from the ascidian *Polycitor* sp., exhibits potent inhibitory capacity against both RNA- and DNA-directed DNA polymerases [28]. Polycitone B and prepolycitrin A were isolated from the marine ascidian *Polycitor africanus* [29].
- Storniamides which are of peptide origin, isolated from the Patagonian sponge *Cliona* sp.
- Ningalins containing from two to five condensed DOPA precursor units [30]. This subgroup includes antioxidant alkaloid purpurone extracted from the Pacific Ocean sponge *Iotrochota* sp. which inhibits ATP-citrate lyase [31], and baculiferins A-O isolated from the Chinese marine sponge *Iotrochota baculifera* and which binds to HIV-1 target proteins [32].
- Lamellarins, with a benzopyrano-pyrrolo-isoquinolinone nucleus, represent the most extensively studied subtype of DOPA-derived marine pyrrole alkaloids. These polycyclic compounds are produced by a variety of organisms, including molluscs, ascidians, and sponges. This large group of marine alkaloids is described further here.

More than 100 such DOPA-derived pyrrole alkaloids have been reported from diverse marine organisms. A wide range of biological activities have been described with these compounds, including cytotoxicity against tumor cells, HIV-1 integrase inhibition, multidrug resistance reversal activity and immunomodulatory activity.

## 2. Structural Diversity of Natural and Synthetic Lamellarins

**Figure 2:**
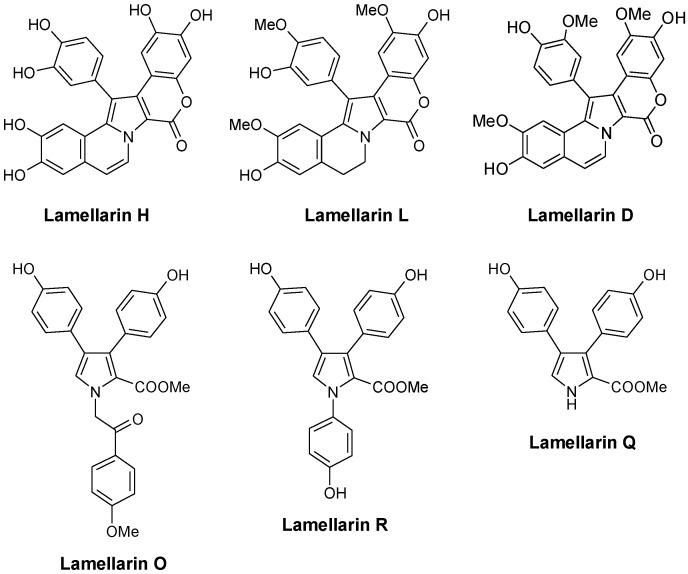
Selected examples of natural lamellarins.

The first compounds in the series, called lamellarins A to D, were identified by Faulkner in 1985 from the Palauan prosobranch mollusc *Lamellaria* sp., a small slug-like sea snail, marine gastropod in the Velutinidae family [33]. The pioneer works of Faulkner [33], Quinn [34], Fenical [35], Boger [36], Ishibashi [37] and others contributed to the discovery and synthesis of new lamellarins and then to the characterization of the modes of action of this group of marine alkaloids. Lamellarin D is without doubt the lead compound in the series, with a mechanism of action largely studied (see below). Many pentacyclic derivatives of lamellarin D have been characterized, such as lamellarins T, U, and V from an unidentified ascidian from the Arabian Sea [38]. In parallel, pyrrole tri-substituted open forms were also discovered, such as Lamellarin O (Figure 2) first isolated from the Australian marine sponge *Dendrilla cactos* [39]. Lamellarins P, Q, and R fall in this group of unfused branched structures, reminiscent of the structures of lukaniols, ningalins and polycitones. Albeit generally less active than the pentacyclic condensed forms, the tri-substituted pyrrole structures are also of interest. Neolamellarin A, a metabolite isolated from the sponge *Dendrilla nigra* and structurally close to lamellarin O, was found to inhibit hypoxia-inducible factor-1 (HIF-1) activation [40,41].

Very quickly, the Latin alphabet was found to be too short to designate all the new lamellarins identified. In 1999, lamellarin-Z was isolated from the Australian ascidian *Didemnum chartaceum*, along with sulfated derivatives of lamellarins B, C, G, and L [34]. Derivatives sulfated at the 20-position, such as lamellarins T-U-V-Y 20-sulfate, are generally not very stable [38]. Following this, the Greek alphabet was used to cite new lamellarins, such as lamellarin α, lamellarin γ, and lamellarin ε, isolated from the Indian Ocean ascidian *Didemnum obscurum* [42,43]. The family of lamellarins rapidly grew to reach 35 members in 2001 and continues to extend, with about 70 members today, including ~50 lamellarins and ~20 related alkaloids with a different name. The most recent natural products are lamellarins A1 to A5 isolated from a *Didemnum* species collected near the Wasp Island, New South Wales [44]. For synthetic derivatives, the most recent publication refers to the preparation of lamellarin η and its dehydro analogue in 10 steps [45]. The term lamellarin now refers to a large family of pyrrole-derived marine alkaloids including more or less extended/condensed structures. These compounds have attracted considerable interest from pharmacologists searching for novel bioactive molecules. They have also considerably engaged the chemistry community with the design of very diverse synthetic analogues, more or less similar to the natural products [46,47]. The literature is rich in procedures to access to heterocyclic lamellarin derivatives. Many synthetic routes have been proposed [48,49]. For example, among the recently described methods, we can cite the assembly of chromenes or benzo-fused chromenes with dimethoxybenzyl-dihydroisoquinolines which generate pentacyclic or hexacyclic derivatives. Annulation of a pyrrole ring to chromene represents a versatile method to generate lamellarin analogues [50]. Efficient routes to the synthesis of lamellarin G trimethyl ether and lamellarin U have also been described [51,52]. By varying the substituents on the pentacyclic core and including a saturated or unsaturated D-ring, a large panoply of natural and unnatural derivatives can be obtained [53]. A new synthesis of lamellarins C and I based on the β-selective C-H arylation of pyrroles has been proposed [54]. There are different routes leading to the pentacyclic skeleton of lamellarins D, L, N, and U, with relatively good yields and versatile approaches (>50%) [55,56,57]. More than 15 total syntheses of lamellarins have been reported. In one of the most concise syntheses, the two pentacyclic lamellarins D and H were both made in seven steps and the simpler, non-fused lamellarin R was made in five steps with a total yield of 53% [58]. The production of diverse lamellarins in a few steps with high yields has been reported, such as the recent modular synthesis of 5,6-saturated lamellarin L and unsaturated lamellarin N via differentially arylated pyrrole-2-carboxylate intermediates [59].

**Figure 3:**
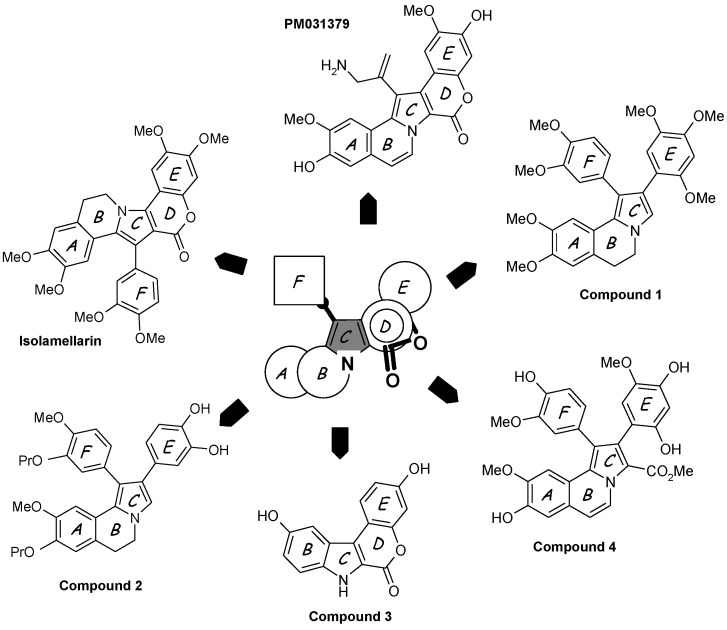
Selected examples of synthetic lamellarin derivatives. The pentacyclic *A*–*E* unit and/or the appended aryl group *F* are modified or truncated. The central pyrrole ring *C* is preserved whereas the lactone *D* ring is often removed or opened.

Among the numerous non-natural lamellarins with a saturated or unsaturated D-ring, one can mention:

(1) Simplified structures lacking the aryl group perpendicular to the pentacyclic core, such as 1-dearyllamellarin D derivatives [60].
(2) Isolamellarin (Figure 3), an isomeric analogue of lamellarin G trimethyl ether [61].
(3) The PharmaMar compound PM031379 (Figure 3), an amino derivative of lamellarin D which induces the nuclear translocation of the apoptosis inducing factor (AIF) in the non-small cell lung cancer cell line U1810 [62]. This synthetic analogue is a potent proapoptotic agent triggering mitochondrial permeability transition via the generation of reactive oxygen species and up-regulation of the apoptosis inducing factor [62].
(4) A diazaindeno[2,1-*b*]phenanthrenone derivative, designed on the basis of molecular modeling of the Lam-D-topoI complex, 100-fold less cytotoxic than Lam-D but maintaining a reduced capacity to inhibit topoisomerase I [63].
(5) Pyrrolo[2,1-*a*]isoquinolines, represented by the open chain derivative 1 (Figure 3), an intermediate in the synthesis of the bioactive lamellarin H [64]. The same authors also described the synthesis of the corresponding 5,6-dihydro pyrrolo[2,1-*a*]isoquinolines [65].
(6) Hybrid structures of lamellarin D and combretastatin A4, such as the dihydropyrroloisoquinoline derivative 2 (Figure 3), which proved to be significantly cytotoxic toward a panel of tumor cell lines [66]. The potential targets of these hybrids, topoisomerase I and/or tubulin, are not known.
(7) Chromeno[3,4-*b*]indoles, as potent inhibitors of the kinase DYRK1A. Molecular modeling suggested that in this case, the compounds bind to the ATP active site of the kinase. In contrast, substitution at the C-3 and C-10 positions afforded a bis-hydroxylated chromenoindole derivative (compound 3 in Figure 3) acting as a topoisomerase I inhibitor and exhibiting a significant cytotoxic potential, but these two activities are apparently not linked; another target may be responsible for the cytotoxic action [67].
(8) Polymeric forms of lamellarin D in order to increase the water solubility of the molecules, with structures incorporating polyethylene glycol (PEG) ester moieties or in the form of PEG-based dendrimers [68]. The same authors also described bioconjugates of lamellarin D including a peptidic nuclear localization signal (Pro-Pro-Lys-Lys-Lys-Arg-Lys-Val-OH) to favor the accumulation of the drug in cell nuclei. A peptide-Lamellarin D conjugate proved to be more than 3-fold more cytotoxic than the parent compound against three human tumor cell lines [69]. PEG-containing polymeric units can also be used as nanocarriers to facilitate the delivery of lamellarins [70].
(9) Mannich derivatives of lamellarin D synthesized in more than 25 steps starting from vanillin and isovanillin. These compounds generally inhibit topoisomerase I and a few of them showed superior cytotoxic activity compared to the parent natural product [71]. Beyond these selected examples, many other derivatives have been reported [72,73,74].
(10) Open-lactone lamellarin analogues such as compound 4 (Figure 3) considered as a potential lead compound. It may be susceptible to partial lactonization under biological conditions, to give lamellarin D [75].

Most of these natural and synthetic lamellarin derivatives have been well characterized from a chemical and structural point of view but their biological activity and mechanism of action remain underestimated or incompletely understood. The only member extensively studied is lamellarin-D (Figure 2) which has been the subject of a significant number of pharmacological studies over the past 12 years. Both anticancer and antiviral effects have been described.

## 3. Anticancer Activities of Lamellarins

**Figure 4:**
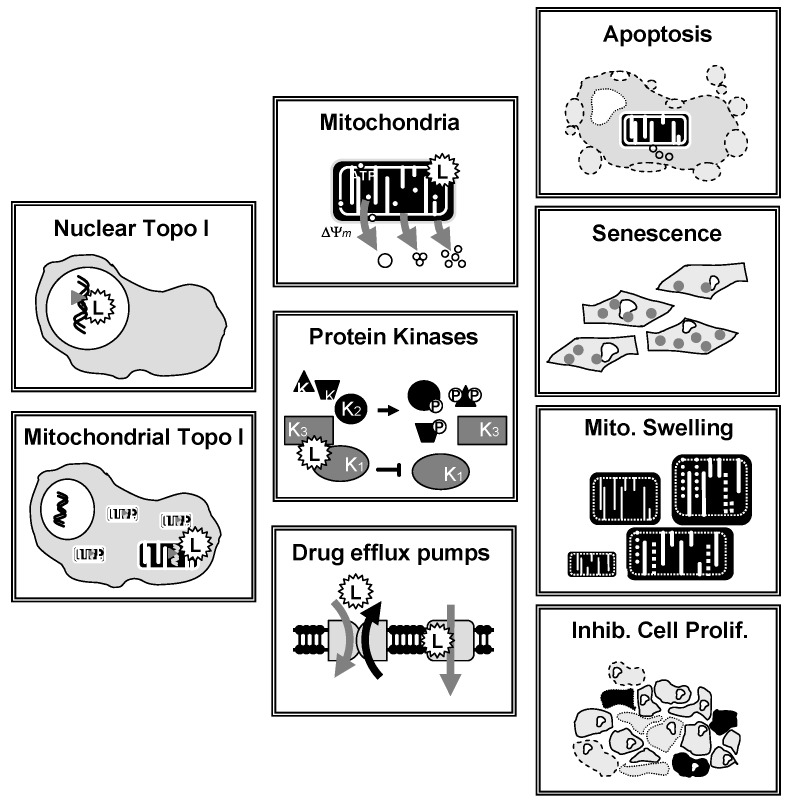
Polypharmacological action of lamellarin D. The drug affects several targets (*stresses*), leading to different cellular effects (*responses*) in cancer cells.

Most lamellarins are potent cytotoxic agents but their capacity to limit the proliferation of cancer cells *in vitro* varies significantly from one compound to another. In general, the condensed penta/hexa cyclic derivatives are much more potent than the trisubstituted pyrrole open forms. Notably, lamellarin D is an extremely potent antiproliferative agent whereas lamellarin O is much less efficient (IC_50_ >10 μM). However, lamellarin O is an inhibitor of the multi-drug resistance efflux pump P-glycoprotein [76] and a selective inhibitor of BCRP-mediated drug efflux [77]. This compound is interesting because it can efficiently reverse Pgp-mediated doxorubicin resistance and BCRP-mediated efflux of mitoxantrone in cancer cells. Structure-activity relationship analyses revealed that the methoxy-acetophenone moiety of lamellarin O is apparently a critical determinant of this BCRP inhibitory activity [77]. The P glycoprotein (P-gp, ABCB1) can limit the anticancer activity, via the efflux of drugs in particular in chemo-resistant cancer cells. Over-expression of P-gp in cancer cells represents a real obstacle to effective chemotherapy for malignant diseases [78]. Interestingly, lamellarins are not particularly sensitive to P-gp. Lamellarin I reverses multidrug resistance by directly inhibiting the P-gp-mediated drug efflux [79]. Similarly, the cytotoxic action of lamellarin D is fully maintained in multidrug-resistant cells compared to a sensitive parental cell line [80]. Lamellarins D, X, ε, M, N, are also among the most potent in the series of natural products [81]. At the molecular level, the mechanism of action of lamellarins remains largely unknown. In 2003, it was discovered that lamellarin D functions as a poison of the topoisomerase I enzyme [82] but it is clear now that a pleiotropic mode of action is responsible for its antiproliferative activity in cancer cells (Figure 4). At least three types of effects have been described, as detailed below.

### 3.1. Inhibition of Topoisomerase I

Twelve years ago, we discovered that lamellarin D was able to strongly promote the conversion of supercoiled DNA into nicked DNA in the presence of topoisomerase I. Like camptothecin, lamellarin D stabilizes topoisomerase I-DNA complexes to induce DNA breaks, but did not induce DNA cleavage by topoisomerase II [82]. P388CPT5 murine leukemia cells resistant to the reference topoisomerase I poison camptothecin are cross-resistant to lamellarin D but the relative resistance index (RRI) is significantly reduced with LAM-D (RRI = 21) compared to CPT (RRI = 103) [80]. This landmark discovery provided the basis to delineate structure-activity relationships in this series. Cell growth inhibition by 41 lamellarin derivatives was evaluated with a panel of tumor cells lines and very clearly the most cytotoxic compounds corresponded to the most potent topoisomerase I poisons [83]. The observed correlation between cytotoxicity and topoisomerase I inhibition indicated that topoisomerase I-mediated DNA cleavage assays can be used as a guide to the development of anticancer agents in this series. Subsequently, a molecular model of the ternary complexes formed between the DNA-topoisomerase I and lamellarin D fully intercalated into the duplex DNA was built and further structural details were defined to help the design of new drugs. The 20-OH and 8-OH of lamellarin D apparently participate in hydrogen-bonding interactions with the side chains of Glu356 and Asn722, respectively, of the enzyme [84]. Based on these studies, different libraries of lamellarins were synthesized and tested for topoisomerase I and cytotoxicity. A few promising compounds were identified [63,75]. Inhibition of topoisomerase I, together with intracellular production of reactive oxygen species, results in the induction of cellular senescence upon treatment with subtoxic concentrations of lamellarin D. The drug can trigger senescence, unlike apoptosis, in the absence of functional mitochondria [85].

Very recently, another fascinating aspect of lamellarin D was identified: its capacity to poison mitochondrial topoisomerase I. In contrast to camptothecin, lamellarin D slows down relaxation of mitochondrial topoisomerase I and strongly inhibits DNA relegation by this mitochondrial enzyme [86]. Mitochondrial topoisomerase I is a genetically distinct mitochondria-dedicated enzyme with a crucial role in the homeostasis of mitochondrial DNA metabolism. This enzyme dampens mitochondrial transcription and thereby alters the respiratory capacity of cells [87]. This key discovery explains, at least partially, the direct mitochondrial effects observed with lamellarin D a few years ago (see below). Cells treated with lamellarin D exhibit dysfunctional mitochondrial respiration, probably as a consequence of the inhibition of mitochondrial topoisomerase I (and other direct effects). Poisoning of mitochondrial topoisomerase I triggers oxidative stress and DNA damage. A link has now been established between the molecular action of lamellarin D on mitochondrial topoisomerase I and the mitochondrial cascade of events (inhibition of respiratory chain, swelling of mitochondrial matrix, *etc.*). Lamellarin D is the first drug to target mitochondrial DNA by trapping mitochondrial topoisomerase I-DNA intermediates.

It is worth mentioning also that lamellarin H has been shown to be active against the topoisomerase of the *Molluscum contagiosum* virus (MCV) [88] and this poxvirus topoisomerase was also found to be inhibited by the cyclic depsipeptide sansalvamide A produced by a marine fungus [89]. Inhibition of topoisomerase I by Lamellarin D plays a significant role in its cytotoxic activity but it is not the unique mechanism of action of this marine compound. Lamellarin D also maintains a marked cytotoxicity toward cell lines resistant to the reference topoisomerase I poison camptothecin, suggesting that another mechanism also contributes to the cytotoxic action [90].

### 3.2. Inhibition of Protein Kinases by Lamellarins

Marine sponges have yielded a great number of compounds that exhibit significant inhibitory activity towards a range of protein kinase [91]. In the course of a screening process, it was discovered that certain lamellarins can interfere with the activity of multiple kinases relevant to cancer, including cyclin-dependent kinase (CDKs) and glycogen synthase kinase-3 (GSK-3). Lamellarin D is a modest kinase inhibitor, with IC_50_ values in the low µM range, whereas lamellarin N proved to be much more potent, with IC_50_ values in the nM range. For example, Lamellarin N was found to be a very potent inhibitor of GSK-3, but also affecting many other kinases to a lesser extent [92]. The kinases inhibition may contribute, at least to some extent, to the cytotoxic and pro-apoptotic properties of lamellarin N. This work opened the way for the design of lamellarin-derived kinases inhibitors, such as chromeno[3,4-*b*]indoles developed as lamellarin isosteres and for which two lead compounds were identified as nanomolar inhibitors a the kinase DYRK1A (dual-specificity tyrosine-(Y)-phosphorylation-regulated kinase 1A), that is a potential drug target for neurodegenerative diseases and cancer [67]. Recently, significant inhibition of protein kinases was reported with the two enantiomers (a*R*)- and (a*S*)- of axially chiral 16-methyl lamellarin N [93]. Interestingly, these two compounds showed no inhibition of topoisomerase I (in contrast to parental lamellarin N) and interfered differently with several kinases. The (a*S*) isomer behaves as a selective inhibitor of kinases GSK-3α/β, PIM1, and DYRK1A, whereas the (a*R*) isomer showed a broader spectrum with non-selective inhibition of many kinases including several cyclin-dependent kinases [93]. However globally, the contribution of kinases inhibition to the anticancer effects of lamellarins still remains largely unclear.

### 3.3. Lamellarin-Induced Mitochondria Perturbations

**Figure 5:**
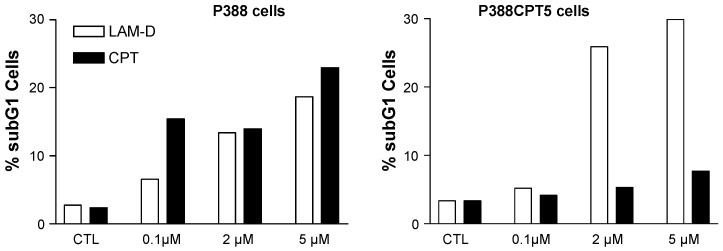
Apoptosis induced by lamellarin D (open bars) and camptothecin (black bars) in murine leukemia cells sensitive (P388) or resistant (P388CPT5) to camptothecin. Apoptosis was measured by cytometry, via the evaluation of the sub-G1 cell population.

Lamellarin D activates two complementary signaling pathways: a nuclear pathway via topoisomerase I inhibition and a direct mitochondria-related pathway which also concurs to trigger cell death. In addition to interfering with topoisomerase I at the nuclear level, lamellarin D directly acts on cancer cell mitochondria to induce apoptosis [90]. The direct mitochondrial effect of lamellarin D accounts for the sensitivity of topoisomerase I-mutated P388CPT5 cells resistant to camptothecin. Unlike camptothecin, lamellarin D induces apoptosis (increase of the sub-G1 cell population) in CPT-resistant cells (Figure 5). Using enucleated cells and cell-free system assays, we were able to show that lamellarin D was targeted to mitochondria to induce apoptosis. In other words, the nuclear targeting seems to be dispensable. The drug can bypass the nucleus to exert a direct action on mitochondria to induce cell death (Figure 4). It induces conformational activation of the pro-apoptotic protein Bax and decreased the expression of the anti-apoptotic proteins Bcl-2 and cIAP2, together with activation of caspases 3 and 9 in leukemia cells. The Fas-dependent extrinsic pathway is not required for lamellarin-D induced apoptosis [94]. The drug can be qualified as a mitochondriophilic agent like betulinic acid and other natural products [95]. The direct action on mitochondria may be an advantage to kill cancer cells resistant to apoptosis (p53 null/mutated cells, topoisomerase 1 mutated cells, Bcl-2 overexpressing cells), providing that a selectivity for cancer cells mitochondria *vs.* normal cells can be delineated. However, this has not been defined as yet. A further study using a highly apoptosis-resistant non-small cell lung carcinoma cell line revealed that lamellarin D and its synthetic amino derivative PM031379 (Figure 3) induced the activation of the Bax protein, the mitochondrial release of cytochrome c and apoptosis-inducing factor (AIF), as well as the activation of caspase-3. However, only the synthetic compound PM031379 triggered cell death and signs of nuclear apoptosis coupled to the nuclear translocation of AIF [62]. Functional mitochondria are required for lamellarin D-induced apoptosis and inhibition of mitochondrial respiration is responsible for apoptosis of cancer cells induced by this compound [96]. These properties make lamellarin D a very interesting compound that may be developed to obtain more effective antiproliferative agents targeting mitochondria for anticancer therapy. However, the difficulty is that mitochondria are also abundant in normal, non-cancer, cells and a selective action is difficult to reach via this central energy-providing mechanism. Nevertheless, this specific action of lamellarin D at the mitochondrial level is very interesting from a mechanistic point of view. How exactly the alkaloid perturbs the mitochondrial metabolism is still a matter of debate but a very elegant metabolomic study suggested that lamellarin D alters the Glu-Asp mitochondrial-cytosolic transport, in particular the malate-aspartate shuttle which involves two tandem-functioning enzymes, aspartate aminotransferase and malate deshydrogenase [97]. Treatment of MCF7 breast cancer cells with lamellarin D induces an accumulation of Glu and Asp metabolites, probably reflecting the inhibition of the malate-aspartate shuttle. This study opens a novel front of research using lamellarin D as a tool to modulate mitochondrial-cytosolic exchanges.

## 4. Other Activities of Lamellarins

Many marine-derived compounds are endowed with antiviral activities, including certain lamellarins [98]. In 1999, Reddy *et al.* [99] showed that lamellarin α 20-sulfate was able to inhibit the integrase activity of the HIV-1 virus, interfering both with the terminal cleavage activity of the enzyme and the strand transfer activity (IC_50_ of 16 and 22 μM, respectively). It also restricted the growth of the HIV-1 virus in cell culture with an IC_50_ value of 8 μM [99]. More recently, the synthesis of sulfated and non-sulfated derivatives of lamellarin α has been successfully completed, enabling the definition of structure-antiviral activity relationships. Interestingly, the sulfated derivatives proved to be active in contrast to the non-sulfated analogue and a ring-opened 20-sulfated analogue, both of which were inactive. Lamellarins α 20-sulfate, 13-sulfate, and 13,20-disulfate were equally active. The key role of the sulfate group was previously established with another natural analogue, lamellarin-ζ, for which the 20-sulfate derivative is active against the HIV-1 integrase, but not the non-sulfated analogue [88]. However, the sulfated derivatives of lamellarin α showed very limited cell uptake capacity, in agreement with their lack of cytotoxic effect. The inaccessibility of the lamellarin sulfates into infected cells suggested that the compounds inhibit HIV-1 infection at the virus entry step rather than at the integration step. The sulfate group is absolutely required to support the anti-viral activity [100].

## 5. Conclusions

**Figure 6:**
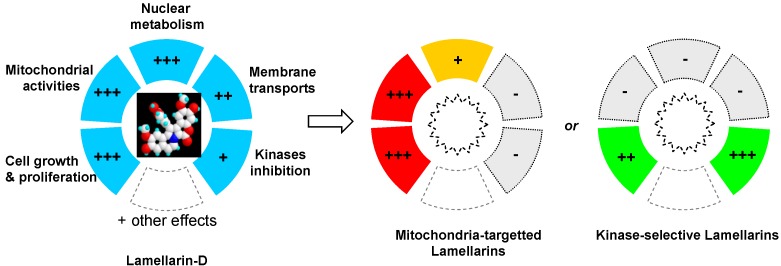
Illustration of the polypharmacological activity of lamellarin D and drug design orientations to synthesize analogues with a more selective mechanism of action.

The development of lamellarin-based drugs with broad-spectrum anticancer activities is a long pursued goal in drug discovery, not yet achieved today. The potent topoisomerase I inhibitor lamellarin D offers an alternative to camptothecin-based drugs, which remain today the only class of topoisomerase I poisons used in the clinic. However, the discovery that lamellarin D not only targets nuclear topoisomerase I but also strongly impacts mitochondrial functions in cells provided a more complex pattern and resulted in a narrower therapeutic index. It is not rare that natural products can modulate multiple targets in cells, which may be an advantage to combat multifactor diseases such as cancer but also requires a complex therapeutic approach. Understanding and exploiting polypharmacology (Figure 6) presents challenges and opportunities for drug discovery [101,102,103]. With the recent key observation that lamellarin D directly interferes with mitochondrial topoisomerase I, in addition to nuclear topoisomerase I, the idea of a next generation of lamellarin derivatives that could selectively impact one cell compartment (nuclear or mitochondrial but not both) may be envisaged (Figure 6). Personally, I believe that targeting both pathways may be an advantage to combat chemo-resistant tumors. Cancers harbor robust biological networks that are inherently resistant to changes and the activities of drugs with a single mechanism of action are often rapidly bypassed by cancer cells. Targeting nuclear topoisomerase I in a camptothecin-like manner is an effective but insufficient mechanism. Targeting mitochondrial topoisomerase I, in addition, may be a new option. There are arguments suggesting that mitochondria could be therapeutic targets of drug resistance in cancer cells [104,105,106]. Mitochondrial metabolic pathways are potential routes to design innovative anti-cancer therapy but at the same time, these pathways play an important role in normal development, maintenance of tissue homeostasis, the regulation of the immune system, and other key functions in normal tissues. The objective would not be to target mitochondria *per se*, but to exert a selective impact on cancer-specific mitochondrial alterations or dysfunctions (e.g., functional alterations, impaired biogenesis, or dynamics). It has even been considered that “the future of medicine will come through mitochondria.” [107]. Perhaps and in this case lamellarins may become useful molecular tools. Over the past twenty years, the vast majority of topoisomerase I inhibitors (mostly camptothecin-based synthetic molecules) developed as anticancer drugs have failed in the clinic, and consequently the enthusiasm for this class of cytotoxic drugs has been attenuated. A re-emergence of topoisomerase I-targeted natural products is probably not viable but the dual action of lamellarin D on nuclear and mitochondrial topoisomerase I poses a new angle to this field of research and raises new questions. A new exploration of lamellarins as anticancer agents is certainly warranted.
